# Is endourological intervention a suitable treatment option in the management of iatrogenic thermal ureteral injury? A contemporary case series

**DOI:** 10.1186/s12894-022-01094-5

**Published:** 2022-09-03

**Authors:** Oğuz Özden Cebeci

**Affiliations:** Department of Urology, Kocaeli Derince Traning and Research Hospital, Saglik Bilimleri University, Ibni Sina Blv 1, 41200 Derince, Kocaeli Turkey

**Keywords:** Iatrogenic, Ureter, Thermal injury, Endourological intervention, Hysterectomy, Laparoscopic surgery

## Abstract

**Background:**

Iatrogenic ureteral injury (IUI) is relatively rare, however, can cause sepsis, kidney failure, and death. Most cases of IUI are not recognized until the patient presents with symptoms following pelvic surgery or radiotherapy. Recently, minimally invasive approaches have been used more frequently in the treatment of IUI. This study evaluates urological intervention success rates and long-term clinical outcomes according to the type of IUI following hysterectomy.

**Methods:**

Twenty-seven patients who underwent surgery due to IUI in our clinic following hysterectomy were evaluated between January 2011 and April 2018. Patients were classified according to the time of diagnosis of IUI. The IUI cases diagnosed within the first 24 h following hysterectomy were designated as "immediate" IUI, while that diagnosed late period was considered 'delayed' IUI. The type of IUI was categorized as "cold transection" if it was due to surgical dissection or ligation without any thermal energy, and "thermal injury" if it was related to any energy-based surgical device. Patient information, laboratory and perioperative data, imaging studies, and complications were assessed retrospectively.

**Results:**

All cases of delayed diagnosis IUI were secondary to laparoscopic hysterectomy (*P* = 0.041). Patients with thermal injury to the ureter were mostly diagnosed late (delayed) (*P* = 0.029). While 31% of the patients who underwent endourological intervention were diagnosed immediately, 69% of them were diagnosed as delayed. These rates were roughly reversed for open reconstructive surgery: 73% and 27% (*P* = 0.041), respectively. We detected eight ureteral complications in our patient cohort following the urological intervention. In all these failed cases, the cause of IUI was a thermal injury (*P* = 0.046) and the patients had received endourological treatment (*P* = 0.005). No complications were detected in patients who undergo open urological reconstructive surgery. While one of the patients who developed urological complications had an immediate diagnosis, seven were in the delayed group (*P* = 0.016).

**Conclusion:**

Endourological intervention is performed more frequently in delayed diagnosed IUI following hysterectomy, however, the treatment success rate is low if thermal damage has developed in the ureter. Surgical reconstruction is should be preferred in these thermal injury cases to avoid further ureter-related complications.

## Background

The ureter runs over the iliac vessels and through the uterine artery into the major pelvis; Thus, it is vulnerable to iatrogenic damage during pelvic surgery.

Owing to the rise in the total number of surgical procedures and the widespread use of minimally invasive surgical methods, the occurrence of iatrogenic ureteral injury (IUI) has increased over the past two decades [1, 2]. Those IUI cases that are not caused by urological surgery are often the result of gynecological surgery [3]. In particular, IUI occurs more frequently in laparoscopic hysterectomies than in the open procedure, as the ureter is harder to identify without tactile and visual cues [4].

The ureter is commonly injured in the lower one-third segment, between the uterine artery and the ureterovesical junction [5].

Early diagnosis and immediate repair can minimize ureter-related complications during long-term follow-up [6], although most cases can be detected in the postoperative period [7]. The location of the traumatized segment and the type of injury are decisive factors in the choice of the surgical approach to treatment [8].

Reconstructive surgeries are recommended for middle and distal ureteral injury [9, 10]. However, recently some studies suggest endourological intervention for first-line treatment of the IUI [11–14]. Those studies have documented success rates across a broad spectrum (17–84%) due to the heterogeneity of IUI etiology, the low density of cases, and the diversity of treatment options [12–17].

To the best of our knowledge, there is no study investigating the treatment outcomes based on the type of injury to the ureter. In this paper, we aim to evaluate urological intervention success rates and long-term clinical outcomes according to the type of IUI following hysterectomy.

## Methods

We retrospectively analyzed the medical records of twenty-seven patients who underwent surgical intervention for IUI in our centers between January 2011 and April 2018. No patients were excluded from the study—all IUI cases were complications of open or laparoscopic hysterectomy.

This retrospective study was approved by the Ethics Committee of Kocaeli Derince Traning and Research and was conducted according to the Ethics Committee of Kocaeli Derince Traning and Research Hospital guidelines. The procedures used in this study adhere to the tenets of the Declaration of Helsinki. Informed consent was obtained from all individual participants included in the study. Collected data were categorized as either gynecological or urological. Gynecological data consists of the patient's age, surgical history, surgical etiology, procedure, histopathological result, and postoperative complication. Collected urological data included the type of ureteral injury, side and location of the injury, time of diagnosis, urological intervention, post-interventional complication, follow-up time, and clinical outcome.

Patients were grouped according to the time of diagnosis of the IUI. While ‘'Immediate' diagnosed ureteral injuries were recognized and repaired at the time of ureteral injury or perioperatively, ureteral injuries recognized a day after hysterectomy or later were classified as 'delayed' IUI similar to the previous studies [18].

Evaluated findings were previous surgery, cause of gynecological surgery, gynecological surgical procedure, time of diagnosis of IUI, urological intervention, and post-urological complication.

The type of delayed diagnosed ureteral injuries was detemined by revisualizing retrospectively video recordings of laparoscopic patient's hysterectomy. For the patients in the immediate IUI group, injury types were defined during gynecological surgery.

The IUI type was categorized as "cold transection injury" if the IUI was due to surgical dissection or ligation and "thermal injury" if any energy-based surgical device caused it. The device causing the thermal damage was Ultracision Harmonic Scalpel® (Ethicon, Cincinnati, OH) in all patients.

Two surgeons performed all urologic interventions. All immediately diagnosed patients were followed up by the same surgeon who performed the urological intervention. The delayed diagnosed group presented with symptoms such as vaginal urine discharge, localized urinoma, renal colic, low urine volume, or pelvic pain. Patients were evaluated either by contrast-enhanced computed tomography urography or intravenous urography. A retrograde urethrography and ureterorenoscopy (4.5 Fr, Richard Wolf, Knittlingen, Germany) were performed for the scope of the ureteral injury.

Patients were treated either endoscopically or with reconstructive surgery, depending on the segment and the extent of the ureteral damage and the surgeon's discretion. An open-end 4.8 F 26 cm ureteral double-j ureteral stent (Coloplast Vortek®) was placed with a 0.035-inch diameter hydrophilic coated guidewire (Cook RoadRunner®) under fluoroscopy for the endoscopically treated patients. These endoscopically treated patients were discharged the same day, following their outpatient surgery. A double-j ureteral stent was removed at the twelve weeks postoperatively for the endoscopic treatment patients.

For the open repair group, ureteroneocystostomy (UNC) was performed using the Lich Gregoir technique. A urethral catheter was placed in the patients of the open repair group for one week. The open surgery group was discharged on the second day postoperatively or later, as appropriate. A double-j ureteral stent was removed at the sixth week postoperatively for open surgery treatment patients.

All patients were scheduled for a follow-up protocol and evaluated with a urinalysis, renal function tests, renal ultrasound, and physical examination bi-annually. Complications after urological intervention were categorized using the Clavien-Dindo classification system [19]. No patients were lost to follow-up. Successful treatment was defined as a stricture-free ureteral function. Prolonged leakage, ureteral stricture, or renal functional loss was considered unsuccessful treatment. The study end-point was defined as long-term clinical outcomes of IUI treatment.

Assumption of Normality assessment was used to test whether or not variables were normally distributed. Descriptive analyses were presented using the mean ± standard deviation or median, the interquartile range (IQR). Fisher's exact test was used to compare categories. All analyses were performed using STATA 14.2 (StataCorp, TX). Statistical significance was set at 0.05, and all tests were two-tailed.

## Results

All cases included in the study consisted of patients in whom the one-third distal segment of the ureter was traumatized secondary to hysterectomy. The IUI was recognized immediately at the time of ureteral injury in 48% (n = 13/27) of the patients and the diagnosis was delayed in 52% of these cases (n = 14/27). Patients in the delayed diagnosis group had undergone laparoscopic hysterectomy (*P* = 0.041) (Table 2). The median time to diagnosis in the delayed diagnosis group was 12 days (IQR 7–20). See Table 1 for a summary of the detailed demographic and clinical characteristics of the patients.

**Table 1:** Demographic and clinical data of the patients

Pts	Age	Previous surgery	Gynecologic procedure	Pathology	Type of Ureteral injury	Side	Complication	Time of diagnosis (day)	Urological intervention (UI)	Complication after UI	Second UI	Complication after the second UI	Follow up (month)	Recent status
1	53	No	LHS	Myoma uteri	Cold transection	R	UVF	7	UNC	No	No	No	44	No Symptom
2	64	No	LHS	Endometrium Carcinoma	Thermal injury	R	UVF	14	DJ placement	No	No	No	42	No Symptom
3	53	No	OHS	Endometrium Carcinoma	Thermal injury	R	Transection (total)	0	UNC	No	No	No	44	No Symptom
4	54	Appendectomy	OHS	Teratoma	Cold transection	R	Transection (partial)	0	DJ placement	No	No	No	36	No Symptom
5	53	No	LHS	Myoma uteri	Thermal injury	R	UVF	1	DJ placement	Stricture	DJ insertion	No	40	No Symptom
6	45	No	LHS	Myoma uteri	Thermal injury	R	UVF	1	UNC	No	No	No	46	No Symptom
7	63	No	LHS	Endometrium Carcinoma	Thermal injury	R	UVF	7	DJ placement	UVF	UNC	No	53	No Symptom
8	37	Myomectomy	OHS	Myoma uteri	Thermal injury	R	Transection (total)	0	UNC	No	No	No	53	No Symptom
9	61	No	LHS	Myoma uteri	Thermal injury	R	Transection (partial)	0	DJ placement	No	No	No	50	No Symptom
10	50	Appendectomy	LHS	Myoma uteri	Thermal injury	R	Ureteral stricture	7	DJ placement	No	No	No	59	No Symptom
11	51	No	LHS	Myoma uteri	Cold transection	R	Transection (partial)	0	DJ placement	No	No	No	43	No Symptom
12	55	No	LHS	Myoma uteri	Thermal injury	R	UVF	20	DJ placement	Stricture	UNC	No	65	No Symptom
13	45	No	LHS	Cervix Carcinoma	Thermal injury	R	Ureteral Stricture	10	DJ placement	Stricture	DJ insertion	Stricture	60	Stricture
14	55	No	LHS	Myoma uteri	Thermal injury	R	Transection (total)	0	UNC	No	No	No	52	No Symptom
15	51	No	LHS	Endometriosis	Cold transection	R	Transection (total)	0	Ureterouretrostomy	No	No	No	50	No Symptom
16	59	No	LHS	Myoma uteri	Thermal injury	R	UVF	21	UNC	No	No	No	52	No Symptom
17	52	Appendectomy	LHS	Myoma uteri	Thermal injury	R	Transection (partial)	0	DJ placement	Stricture	UNC	No	57	No Symptom
18	56	No	OHS	Endometriosis	Cold transection	R	Transection (partial)	0	DJ placement	No	No	No	44	No Symptom
19	41	No	LHS	Myoma uteri	Thermal injury	R	Transection (partial)	0	Ureterouretrostomy	No	No	No	43	No Symptom
20	48	No	LHS	Uterine bleeding	Thermal injury	L	UVF	16	DJ placement	No	No	No	46	No Symptom
21	47	Appendectomy	LHS	Cervix Carcinoma	Thermal injury	L	Transection (total)	0	UNC	No	No	No	10	No Symptom
22	57	No	LHS	Cervix Carcinoma	Thermal injury	L	UVF	7	DJ placement	No	No	No	6	No Symptom
23	52	No	LHS	Myoma uteri	Thermal injury	L	UVF	21	DJ placement	Stricture	UNC	Stricture	6	Stricture
24	47	No	LHS	Endometriosis	Cold transection	L	Transection (total)	0	Ureterouretrostomy	No	No	No	2	No Symptom
25	50	No	LHS	Myoma uteri	Cold transection	L	Transection (total)	0	Ureterouretrostomy	No	No	No	64	No Symptom
26	64	No	LHS	Endometrium Carcinoma	Thermal injury	L	Ureteral Stricture	30	DJ placement	Stricture	DJ insertion	Stricture	72	Stricture
27	45	No	LHS	Myoma uteri	Thermal injury	R	UVF	20	DJ placement	Stricture	UNC	No	69	No Symptom

*LHS* laparoscopic hysterectomy, *OHS* Open hysterectomy, *UVF* ureterovaginal fistula, *UNC* Ureteroneocystostomy

Immediate diagnosis was made in 86% (n = 6/7) of the patients whose ureteral injury was due to cold transection injury. However, in patients with delayed diagnosis, IUI mostly developed secondary to a thermal injury (*P* = 0.029) (Table 2). Of the patients who underwent endourological intervention, 31% (n = 5/16) were diagnosed immediately and 69% (n = 11/16) were diagnosed as delayed. In comparison, in patients who underwent open reconstructive surgery, these rates were observed to be 73% (n = 8/11) and 27% (n = 3/11), respectively (*P* = 0.041) (Table 2).

**Table 2:** Diagnosis time and clinical parameters

	Immediate diagnosis (n = 13)	Delayed diagnosis (n = 14)	*P-* value
*Previous surgery n (%)*			0.140
No surgery	9 (40.9)	13 (59.1)	
Abdominal surgery	4 (80)	1 (20)	
*Cause of gynecologic surgery n (%)*			0.564
Malign + Endometriosis	5 (45.45)	6 (54.55)	
Benign	8 (50)	8 (50)	
*Gynecologic procedure n (%)*			0.041
Abdominal surgery	4 (100)	0	
Laparoscopic Surgery	9 (39.13)	14 (60.87)	
*Type of ureteral injury n (%)*			0.029
Cold transection	6 (85.71)	1 (14.29)	
Thermal injury	7 (35)	13 (65)	
*Urological intervention n (%)*			0.041
Endoscopic	5 (31.25)	11 (68.75)	
Reconsturictive surgery	8 (72.72)	3 (27.28)	
*Complications of after urological interventions n (%)*			0.021
No	12 (63.15)	7 (36.85)	
Yes	1(12.5)	7 (87.5)	

According to the Clavien-Dindo classification system, we detected eight grade 3b ureteral complications in our patient’s cohort. In all of these eight cases, IUI was due to thermal injury (*P* = 0.046), and their first urological intervention was endoscopic double-j ureteral stenting (*P* = 0.005) (Table 3). One of these patients was diagnosed immediately, and seven were delayed (*P* = 0.016) (Table 3). The ureteral stricture was developed in six of these seven patients, and a ureterovaginal fistula was seen in one (Table 1).

**Table 3:** Factors affecting urological complications

	Recovery after urological intervention (n = 19)	Complication after urological intervention (n = 8)	*P-value*
*Previous surgery n (%)*			0.601
Yes	4 (80)	1 (20)	
No	15 (68)	7 (32)	
*Cause of gynecologic surgery n (%)*			0.824
Benign	11 (69)	5 (31)	
Malign + Endometriosis	8 (73)	3 (27)	
*Gynecologic procedure n (%)*			0.160
Abdominal hysterectomy	4 (100)	0	
Laparoscopic hysterectomy	15 (65)	8 (35)	
*Type of ureteral injury n (%)*			0.046
Cold transection	7 (100)	0	
Thermal injury	12 (60)	8 (40)	
*Time of diagnosis n (%)*			0.016
Perioperative	12 (92)	1 (8)	
Delayed	7 (50)	7 (50)	
*First Urological intervention n (%)*			0.005
Endoscopic	8 (50)	8 (50)	
Reconstructive surgery	11 (100)	0	

Lich Gregoir ureteroneocystostomy was performed in five of these eight patients, and no postoperative complications occurred in the follow-up. The remaining three patients with ureteral stricture did not consent to open or laparoscopic ureteral reimplantation. These patients were followed up with repetitive ureteral dilatations and double-j ureteral stenting to protect the renal unit. No renal dysfunction or hydronephrosis was observed at a median follow-up of 58.5 months (IQR 46.5–67) (Table 1).

## Discussion

Our research has revealed that the type of ureteral injury is a crucial factor for urological intervention decisions and treatment efficacy in IUI following hysterectomy. We observed that endourological interventions were performed more frequently in delayed diagnosed IUI cases, and half of these procedures were failed in our patient group. The cause of IUI in those delayed diagnosed patients was mostly thermal injury.

IUI incidence has increased in the past twenty years due to the rise in the overall number of surgeries and the widespread use of minimally invasive surgical techniques [1, 2]. The most common causes of ureteral trauma are suture ligation, blunt injury, partial/total transection, and ischemia due to thermal damage [20].

It is essential to choose the appropriate treatment in IUI. Early detection of trauma and immediate ureteral correction surgery reduces kidney and ureter-related complications [6, 21]. Sepsis (odds ratio: 11.9), urinary fistula (odds ratio: 23.8) and mortality (odds ratio: 1.4) are more common in delayed-diagnosed IUI cases compared to early-diagnosed patients [18]. Approximately three-quarters of IUI malpractice litigation ends up with a decision against the surgeon(s). The most common accusations are prolonged urinary leakage, delayed ureteral reconstruction, inattentive postoperative care, and insufficient surgical training [22].

Recommended treatment modalities in early diagnosed IUI are ureteroureterostomy or ureteral reimplantation, depending on the location of the traumatic ureteral segment [7]. In the present study, all of the patients who underwent reconstructive surgery recovered completely in long-term follow-up. The majority of these cases were consisting of immediately diagnosed patients (Table1).

Since the traumatic ureteral segment is removed in reconstructive surgeries, high treatment success rates are reported even with novel minimally invasive approaches [10, 23]. However, there is no consensus on the initial treatment modality in delayed-diagnosed IUI [24]. ‘Endourological treatment of delayed-diagnosed ureteral injuries by internal stenting, with or without dilatation, is the first step in most cases depending on the nature, severity, and location of the injury site’ [9]. Minimally invasive approaches are often chosen as initial care in the management of IUI due to their less invasive nature, short operative time, short length of hospital stay, fewer complications, and low treatment cost [25].

We diagnosed 86% of patients with cold transection IUI immediately. This rate was 35% in thermal injuries, statistically significantly lower than cold transection (Table 2). Almost one-third of the thermal injuries could not be recognized in early settings in our patient cohort, supporting previous studies [26]. We initially treated 59% of the patients with endourological methods via retrograde fashion similar to the previous studies [14]. Although ureteroscopic ureteral realignment with stenting was successful in all these patients, following double-j ureteral stent removal, our success rate was 50%. In all of these failure cases, ureteral damage was of thermal origin (Table 3). According to current data, a wide range of success rates are reported in endoscopic IUI management (17–84%) though, in most IUI studies, the type of ureteral injury was not specified [12–17, 27]. Our results are similarly revealed a higher amount of re-intervention for the delayed diagnosed patient group (Table 3). More repetitive urological interventions may be required in delayed diagnosed cases [28].

There is a limited number of publications investigating IUI due to thermal damage [29]. Surgical energy devices are known to induce varying degrees of thermal injury to all tissue types [30]. Tissue coagulation devices that work with ultrasonic-based energy vary depending on the device's technical features but can cause an increased temperature between 33 and 100 °C on the surrounding tissue [31] and lateral spread may be up to 10 mm. In ultrasonically activated electrocautery, the temperature rises very quickly to 350 °C, and the lateral distance can reach 22 mm [32]. These tissue heat quantities are higher than 60 °C, even at 25 mm from the device. Therefore, it has been shown that the ultrasonic electrocoagulation tip of the devices causes significant histological damage in thin-walled organs such as the ureter, damage that cannot be detected macroscopically [26]. Heat damage begins to appear when the temperature rises above 45 °C. Protein denaturation and cell death occur as thermal exposure in tissues increases [33]. These deleterious changes cause myofiber atrophy and fibrosis at traumatic tissue margins [34], and eventually, mucosal stenosis may occur [35].

We believe high complication rates are observed in patients treated with endourological intervention following post-hysterectomy thermal IUI because the traumatic ureter segment was not excised in this study. Although the endoscopic surgical method did not fail in the early period, we observed complications due to the ureter in half of the patients during follow-up. Therefore, in our opinion, surgical techniques in which the traumatized segment of the ureter is excised should be preferred in suspected thermal IUI cases to avoid complications such as stricture or fistula.

The limitations of this study are its retrospective design due to IUI cases characteristics, analysis including small sample size, and the lack of knowledge of the energy setup of energy-based surgical instruments used during ureter dissection.

## Conclusions

Early recognition of thermal IUI following hysterectomy and the success rates of endourological intervention are limited. Excision of the traumatized segment and providing ureteral continuity through reconstructive surgery increase the success of surgical treatment in delayed diagnosed thermal IUI.

## Data Availability

The datasets used and/or analyzed during the current study are available from the corresponding author on reasonable request.

## Abbreviations

LHS: Laparoscopic hysterectomy
OHS: Open hysterectomy
UVF: Ureterovaginal fistula
UNC: Ureteroneocystostomy
